# Changes in Physical and Water Sorption Characteristics of Three Solid Woods after One-Sided Surface Charring

**DOI:** 10.1021/acsomega.4c01110

**Published:** 2024-06-17

**Authors:** Carolina Tenorio, Roger Moya, Lidier Tencio

**Affiliations:** Escuela de Ingeniería Forestal, Instituto Tecnológico de Costa Rica, Apartado 159-7050, Cartago, Costa Rica

## Abstract

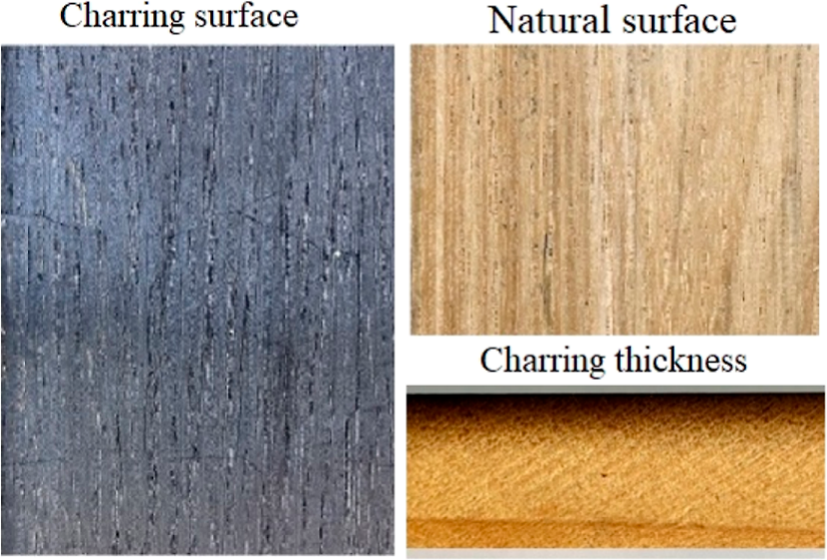

One-sided surface charring of wood is a modification process used to lower moisture absorption and improve the resistance to biological degradation for durable surface exterior claddings. *Cupressus lusitanica*, *Gmelina arborea,* and *Tectona grandis* wood samples from fast-growth plantation were charred with a hot plate using three temperatures (300, 350, and 400 °C ± 3 °C) for 10 min. Wood density, surface quality (color and presence of splits), and sorption characteristics (wetting rate and water uptake) were evaluated. Results show that samples charred at 300 °C presented a lower loss of density and thickness than samples charred at 400 °C. Changes in the chemical structure of the wood as a result of the high temperatures caused a decrease of all color parameters (L*, a*, and b*). These values decreased in the samples charred at 400 °C for the three species. Also, the presence of cracks and splits on the surface, or in some cases the presence of detachments from the charring surface, was mostly observed in the samples charred at 350 and 400 °C. One-sided surface charring reduced the liquid water sorption of wood samples in comparison with that of reference samples, especially for *C. lusitanica* and *T. grandis*. *G. arborea*, due to the composition of its anatomical structure and its initial density, chars faster than the other species, causing a greater loss of density, wetting rate values like those of the reference wood, and higher values of water uptake.

## Introduction

Wood is widely used as a construction material due to its biological origin, wide availability, relatively simple and easy workability, and high strength in relation to its weight.^1^ However, wood is susceptible to weathering degradation caused by ultraviolet radiation and variations in temperature and relative humidity under conditions of use. In addition, wood presents low dimensional stability since it is affected by changes in temperature and relative humidity.^2^ Weathering causes erosion of the wood surface and can lead to further damage caused by biological factors such as fungal activity, while changes in dimensional stability leads to cracking in the wood surface.^3^

Currently, there is an increase for outdoor wood products with high durability to weathering and other biological agents, low maintenance costs, and a homogeneous and pleasant appearance.^2^ Many of these requirements are extremely challenging for standard wood products to meet. To protect wood when used outdoors, it must be coated or modified, which increases the environmental burden and costs not only of investment but also of maintenance.^4^ Furthermore, increasing restrictions on the use of efficient chemicals for protection and durability improvement decrease the competitiveness of wood products.^5^ To solve these disadvantages, a series of more environmentally friendly processes have recently been implemented; chemical modification (e.g., acetylation), impregnation (e.g., furfurylation), and different types of thermal modification processes can be used to lower moisture absorption, and consequently, better dimensional stability and resistance to biological degradation can be reached.^6−9^

Wood modification methods also tend to be quite time-consuming, which increases the cost of the product.^9−11^ Given this series of limitations, as an option, the wood could be modified only from the exposed surface, saving time and costs and preserving the structural properties of the wood.^12^ Charring as a protective treatment for wood is a typical pyrolysis process.^13^ When wood is subjected to pyrolysis, the most reactive components of the wood, that is, the hemicelluloses and the amorphous zones of the cellulose, are degraded, causing a reduction of the hydroxyl groups in these wood components, leading to a decrease in the moisture absorption capacity of wood.^14,15^ At the same time, the pyrolysis process can degrade the hydrophilic hydroxyl groups of wood matrices and increase the hydrophobic groups, improving the dimensional stability of wood.^15^ Furthermore, when the charring of wood components is carried out at high temperature, it significantly reduces the nutrients in the wood, which strongly inhibits the growth of fungi in the wood to achieve resistance to biodeterioration.^16,17^

The surface charring of wood is an ancient Japanese technique called “Shou Sugi Ban” or “Yakisugi” that is used to increase the durability and sustainability of wood.^18^ The process consists of charring the surface of the wood using a flame.^11^ Advances in the charring process have allowed the development of contact heating systems using a heating plate, which allows greater control of temperature conditions, creating a uniform surface and making it a more environmentally sustainable process.^11^ In the process, the wood is pressed against a heated surface for a given modification time.^19^ According to Sandberg et al.,^9^ the depth of the treatment has a major influence on the performance of the product and affects certain properties, such as sorption, cracking during weathering, and thermal insulation. Kymäläinen et al.^7^ described the surface charring by using a high-temperature hot plate and applying a weight on the top to prevent structural deformation. They showed that the protective properties of charring were influenced by wood species, charring temperature and time, and treatment uniformity. Čermák et al.,^20^ for their part, studied the characteristics of wood charred at 220 °C at different times, 15 and 40 min, and demonstrated that charring improved moisture-related characteristics and led to better mechanical properties. Also, Kymäläinen et al.^19^ evaluated the effect of modification time and wood species and density for contact charred wood, and they found that wood density influences the cracking in the surface, char depth, and charring rate.

*Cupressus lusitanica*, *Gmelina arborea,* and *Tectona grandis* from fast-growth plantation have been widely studied in terms of their properties and in thermal and chemical modification processes in Costa Rica.^21−24^ However, the surface charring process has been developed only to achieve artisanal finishes on furniture or coatings but without the technological and scientific development required to carry out the process on a larger scale. Thus, the aim of this study is to investigate the effect of one-sided charring of the surface by using a heating plate with three temperatures on wood density, color, and water sorption characteristics (wetting rate and water uptake) of *C. lusitanica*, *G. arborea,* and *T. grandis* wood.

## Methodology

### Preparation of Wood Samples

Wood samples of *C. lusitanica*, *G. arborea,* and *T. grandis* from fast-growth plantations in the provinces of Cartago, Alajuela, and Guanacaste in Costa Rica were used. Samples of 300 mm length × 70 mm wide × 20 mm thickness, with two grain orientations (radial and tangential), were prepared from each species. The samples were conditioned at 65% relative humidity (RH) and 20 °C temperature (approximately 12% in equilibrium moisture content) before surface charring treatment.

### Surface Charring Treatment

Surface charring was conducted between two metal plates, but only one side (bottom) was heat. The samples were placed between two metal plates, and the bottom plate was heated with three target temperatures 300, 350, and 400 °C ± 3 °C for 10 min (Table 1), with a weight of 10 kg applied on the top plate (surface pressure approximately of 0.073 N/mm^2^) with the objective to reduce deformation. A total of 60 samples per species were one-sided surface charred [3 temperatures × 2 grain orientations (radial and tangential) × 10 samples]. Also, 20 more samples (10 radial and 10 tangential) per species were used as the reference.

**Table 1:** Treatments of One-Sided Surface Charring

temperature(°C ± 3 °C)	time (min)	grain orientation	code
300	10	radial	300-R
		tangential	300-T
350	10	radial	350-R
		tangential	350-T
400	10	radial	400-R
		tangential	400-T

### Physical Properties of One-Side Surface Charred Wood

**Figure 1:**
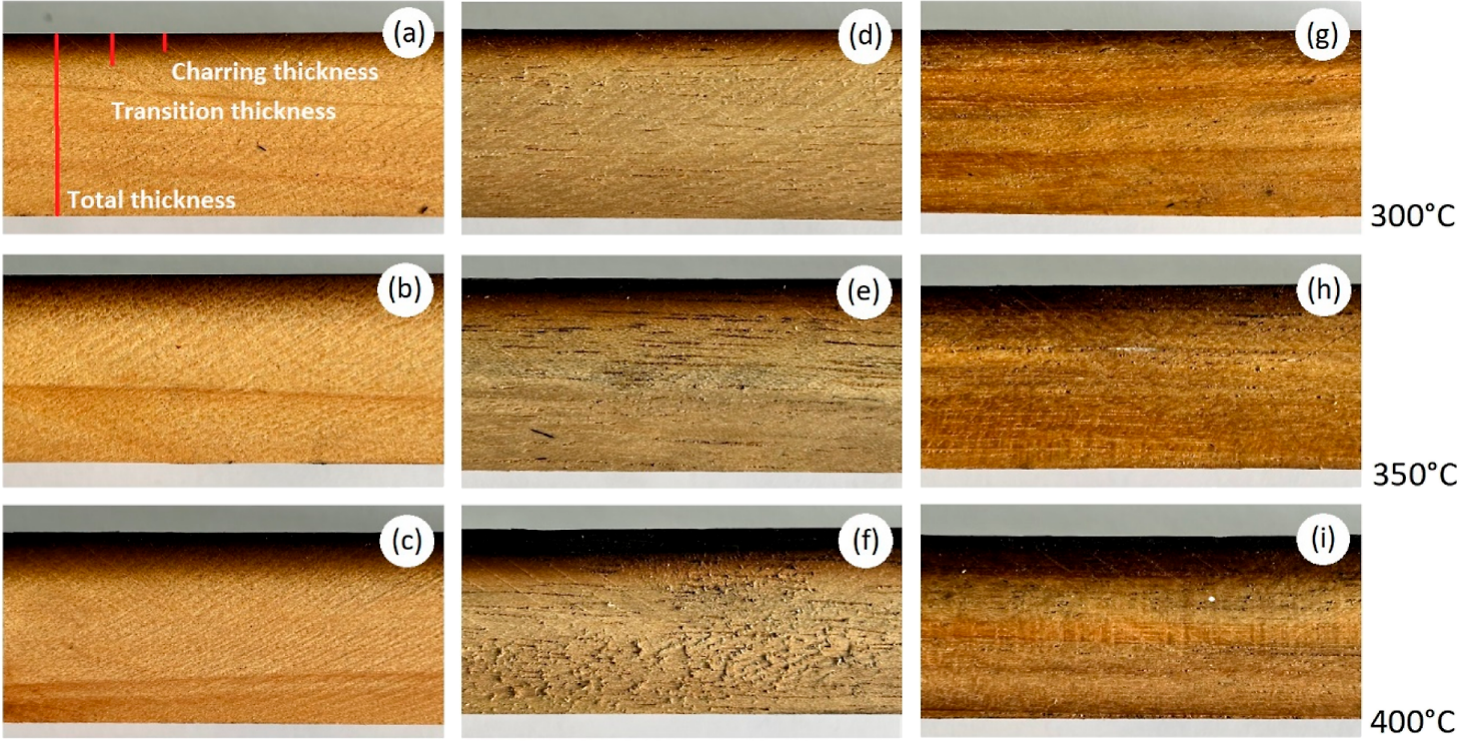
Samples cuts from charred surface wood of *C. lusitanica* (a–c), *G. arborea* (d–f), and *T. grandis* (g–i). Photograph of Carolina Tenorio Monge.

Before and after the surface charring treatment, all of the wood samples were weighed, and their dimensions (width, thickness, and length) were measured. The initial density was calculated before the one-sided surface charred process, and the final density after the process was calculated as the ratio between weight and volume. The initial moisture content was calculated by the ratio of the initial weight (before surface charring) and oven-dry weight (after surface charring), expressed as a percentage according to ASTM D-4422.^25^ In addition, charring thickness, transition thickness, and Δ*T*hickness were determined. The thickness of the charred zone (charring thickness) and the transition zone were determined, as shown in Figure 1. The depth of these parameters was measured using a light stereoscope and a simple rule in mm. The percentage of thickness variation (Δ*T*hickness) was determined by the relationship of the total thickness of the sample before and after surface charring and was expressed as a percentage.

For each species, 10 samples by reference and surface charred treatment with dimensions of 20 mm × 10 mm × 1.5 mm were analyzed for their density profiles. Weight, length, width, and thickness were determined for each sample. The density was measured at intervals of 0.1 mm through the thickness of the samples by using an X-ray densitometer QMS, Model QDP-01 (Quintek Measurement Systmes, Inc., Knoxville, TN). The density profile was measured with respect to the sample thickness.

### Quality of One-Sided Surface Charred Wood

Quality evaluation of the one-sided charred wood was carried out using two parameters: visual inspection and determination of the color change of the wood surface after the charring process. The visual inspection was evaluated with the naked eye, and cracks and splits were observed in the surface. Wood color was measured using a Miniscan XE plus colorimeter (Hunterlab 1995) under room conditions at the Wood Properties Laboratory in the ITCR (±2 in temperature). The colorimeter was calibrated each time it was used using a white standard reference supplied by the company. The reflectance spectra were recorded according to the standardized CIELab’s chromaticity system. The measurement was set within the visible range of 400–700 nm at intervals of 10 nm with a measuring aperture of 11 mm. For the reflection reading, the observer component was set at an angle of 10° to the sample’s normal surface. The light source D65 (corresponding to daylight at 6500 K) was used as a color space measuring and computing parameter. According to HUNTERLAB (1995), the CIELab’s color system estimates the wood color in three coordinates: L* for lightness that represents the position on the black–white axis (*L* = 0 for black and *L* = 100 for white), a* for the chroma value that defines the position on the red–green axis (+100 values for red and −100 values for green), and b* for the chroma value that defines the position of the yellow–blue axis (+100 values for yellow and −100 values for blues). The colorimeter was placed on each of the sample charring surfaces before and after the process.

The color change after charring treatment (ΔE*) was determined and was calculated by the distance between two points in the color coordinate from the split-up values ΔL*, Δa*, and Δb*.

### Sorption Properties of One-Sided Surface Charred Wood

For each surface charred treatment, reference, and species, 10 samples of 100 mm × 50 mm × 20 mm were used for water uptake measurements. All samples were sealed from five sides with an epoxy resin catalyzed and were preconditioned at 65% RH and 20 °C, after which they were set to float face down, so that the charred side was facing downward, in a container with water. The mass was measured after 0, 24, 48, and 72 h, after which the samples were oven-dried for moisture content determination. Moisture uptake was measured from the increase in mass after each step and converted to g/m^2^. Before weighing, any excess water on the surfaces was blotted with paper tissue.

One sample of 28 mm × 11 mm × 11 mm of each surface charred treatment, reference, and species were used to determined wettability by using contact angle measurements. The sample used was intended to present a homogeneous and representative surface of the entire one-sided charring surface of the piece. A small droplet (2 μL) on a charring surface was measured at 20 °C according to the method of Bachmann et al.^26^ The water repellency of the material was measured by placing a small water droplet on the charring surface and recording the contact angle every 10 s during 1200 s (20 min) using an automated goniometer ramé-hart model 590, ramé-hart instruments co, NJ, USA, with DROPimage software 2.5.02 by Finn Knut Hansen, OS, Norway, 2006. Two contact angles were measured, that is, the initial contact angle (θ_initial_) and the contact angle at 20 min (θ_20_). Afterward, the wetting rate was calculated as the variation of the contact angle (θ_20_ – θ_initial_) over 20 min of wetting to assess the spreading and penetration of pure water.

### Statistical Analysis

The statistical analysis confirmed the normality of the results. One-way ANOVA was carried out by means of the GLM procedure of the SAS software (SAS Institute, Campus Drive Cary, NC) to confirm the effect of the grain orientation on the different characteristics in surface charred wood (moisture content, initial and final density, charring and transition thickness, Δ*t*hickness, and color parameters) The Tukey test was used to determine the statistical differences between the means of the variables measured. The analysis of variance and Tukey tests were performed with SAS software (SAS Institute Inc., Cary, NC).

## Results

### Physical Characteristics

*C. lusitanica* and *G. arborea* presented an initial moisture content (MCi) of around 14% on average, while *T. grandis* presented an average close to 13% (Table 2). Some statistical differences at the level of grain orientation were observed in the three species; in the case of *C. lusitanica,* differences were observed at the three charring temperatures (Table 2). However, for *G. arborea* and *T. grandis,* the differences were observed at temperatures of 300 and 400 °C (Table 2).

**Table 2:** Physical Properties and Thickness Variation of the Charring Surface of Radial and Tangential Wood of *C. lusitanica*, *G. arborea,* and *T. grandis*^a^

species	temperature	grain orientation	moisture content (%)	initial density (g/cm^3^)	final density (g/cm^3^)	charring thickness (mm)	transition thickness (mm)	Δ*T*hickness (%)
*C. lusitanica*	300 °C	radial	14.16^B^	0.52^A^	0.50^A^	0.28^B^	1.65^B^	0.33^A^
		tangential	15.34^A^	0.47^B^	0.45^B^	0.57^A^	1.98^A^	0.88^A^
	350 °C	radial	13.43^B^	0.52^A^	0.47^A^	0.54^B^	2.61^B^	0.65^A^
		tangential	14.55^A^	0.52^A^	0.48^A^	0.72^A^	3.25^A^	0.51^A^
	400 °C	radial	12.85^A^	0.52^A^	0.46^A^	1.31^A^	3.33^A^	1.51^A^
		tangential	13.63^B^	0.50^A^	0.44^A^	1.46^A^	3.79^A^	1.23^A^
*G. arborea*	300 °C	radial	14.86^A^	0.43^A^	0.41^A^	0.41^A^	1.85^A^	0.37^A^
		tangential	13.42^B^	0.45^A^	0.42^A^	0.39^A^	1.95^A^	0.32^A^
	350 °C	radial	14.32^A^	0.45^B^	0.40^A^	0.93^B^	2.17^B^	0.26^A^
		tangential	13,51^A^	0.48^A^	0.43^A^	1.30^A^	3.06^A^	1.06^A^
	400 °C	radial	15.53^A^	0.43^A^	0.37^A^	1.92^A^	2.53^A^	2.25^A^
		tangential	13.71^B^	0.47^A^	0.40^A^	1.74^A^	2.74^A^	1.76^A^
*T. grandis*	300 °C	radial	13.61^B^	0.67^A^	0.63^A^	0.43^A^	1.50^A^	0.03^A^
		tangential	12.54^A^	0.65^A^	0.62^A^	0.34^A^	1.47^A^	0.28^A^
	350 °C	radial	12.46^A^	0.55^B^	0.51^B^	0.82^A^	2.45^A^	0.22^A^
		tangential	12.43^A^	0.65^A^	0.61^A^	0.62^A^	1.71^A^	0.72^A^
	400 °C	radial	12.91^A^	0.58^B^	0.52^B^	1.29^A^	3.56^A^	0.51^B^
		tangential	12.08^B^	0.64^A^	0.57^A^	1.36^A^	2.95^A^	1.25^A^

a Note: different letters for each parameter represent statistical differences between thev grain orientation (radial and tangential) for each temperature (significance of 0.05).

For the three species, a slight decrease in density was observed after the one-sided charred surface process for all treatments, and it was observed that the higher the process temperature, the greater the loss in density for the three species (Table 2). In the case of *C. lusitanica*, the density varied from 0.51 to 0.47 g/cm^3^, which represented a loss of 8.8% in density, and statistical differences were observed at the grain orientation level only for the temperature of 300 °C. For *G. arborea,* the density decreased from 0.45 to 0.41 g/cm^3^, which represented a loss of 10.4% in density, and differences were presented at the temperature of 350 °C for the initial density. Finally, for *T. grandis,* the density decreased from 0.62 to 0.58 g/cm^3^ (7.7% loss in density), where the temperatures of 350 and 400 °C presented statistical differences (Table 2).

Regarding the charring thickness and the Δ*t*hickness, it was observed that *G. arborea* samples presented the highest values, followed by *C. lusitanica* and *T. grandis*, while for the transition thickness, *C. lusitanica* presented the highest values and *T. grandis* the lowest values (Table 2). And as expected, both thicknesses increased with increasing charring temperature (Table 2). In *C. lusitanica* samples, differences were observed at the level of grain orientation for the temperatures of 300 and 350 °C for both thicknesses, and for *G. arborea, differences were observed* at the temperature of 350 °C. Also for both thicknesses, the surface charred samples of *T. grandis* did not present any differences at the level of grain orientation (Table 2). Regarding the Δ*T*hickness, it was observed that the higher the process temperature, the greater the Δ*T*hickness for the three species (Table 2). However, statistical differences were presented only at the level of grain orientation at the temperature of 400 °C for *T. grandis* (Table 2).

**Figure 2:**
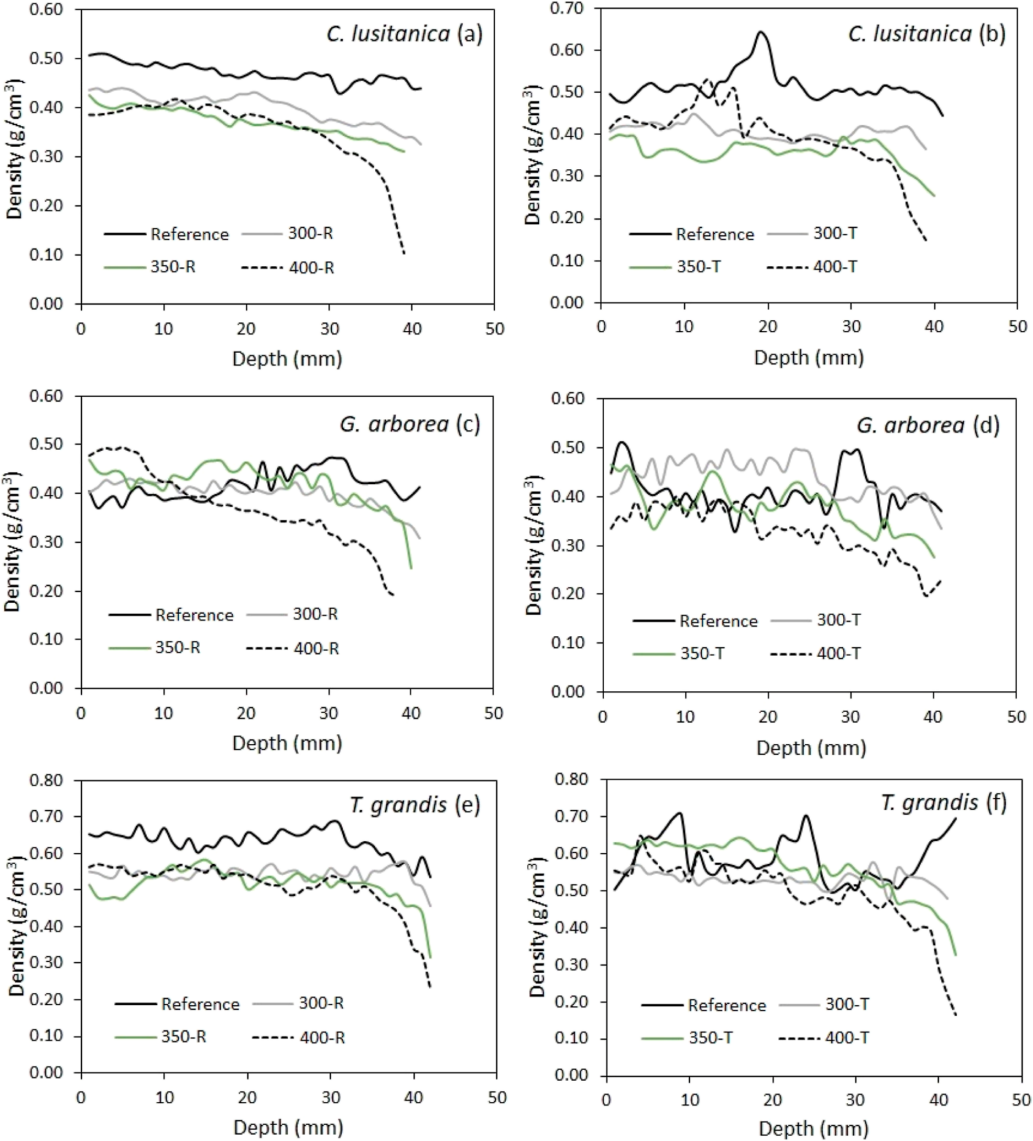
Density profiles in the reference and surface charred radial and tangential wood of *C. lusitanica* (a,b), *G. arborea* (c,d), and *T. grandis* (e,f).

Figure 2 presents the X-ray density profiles for the one-sided surface charring of the radial and tangential samples compared with the reference. Differences were observed between the curves of the density profiles according to the grain orientation; the radial specimens present more uniform curves (Figure 2a,c,e), while the tangential specimens present a greater variation in the density curves for the three species (Figure 2b,d,f). For both radial and tangential specimens after one-sided charring, the density profile decreased according to the temperature applied; the samples charred at 400 °C present a greater decrease in density. However, this behavior is better observed in the radial specimens (Figure 2).

### Quality of One-Sided Surface Charred Wood

**Figure 3:**
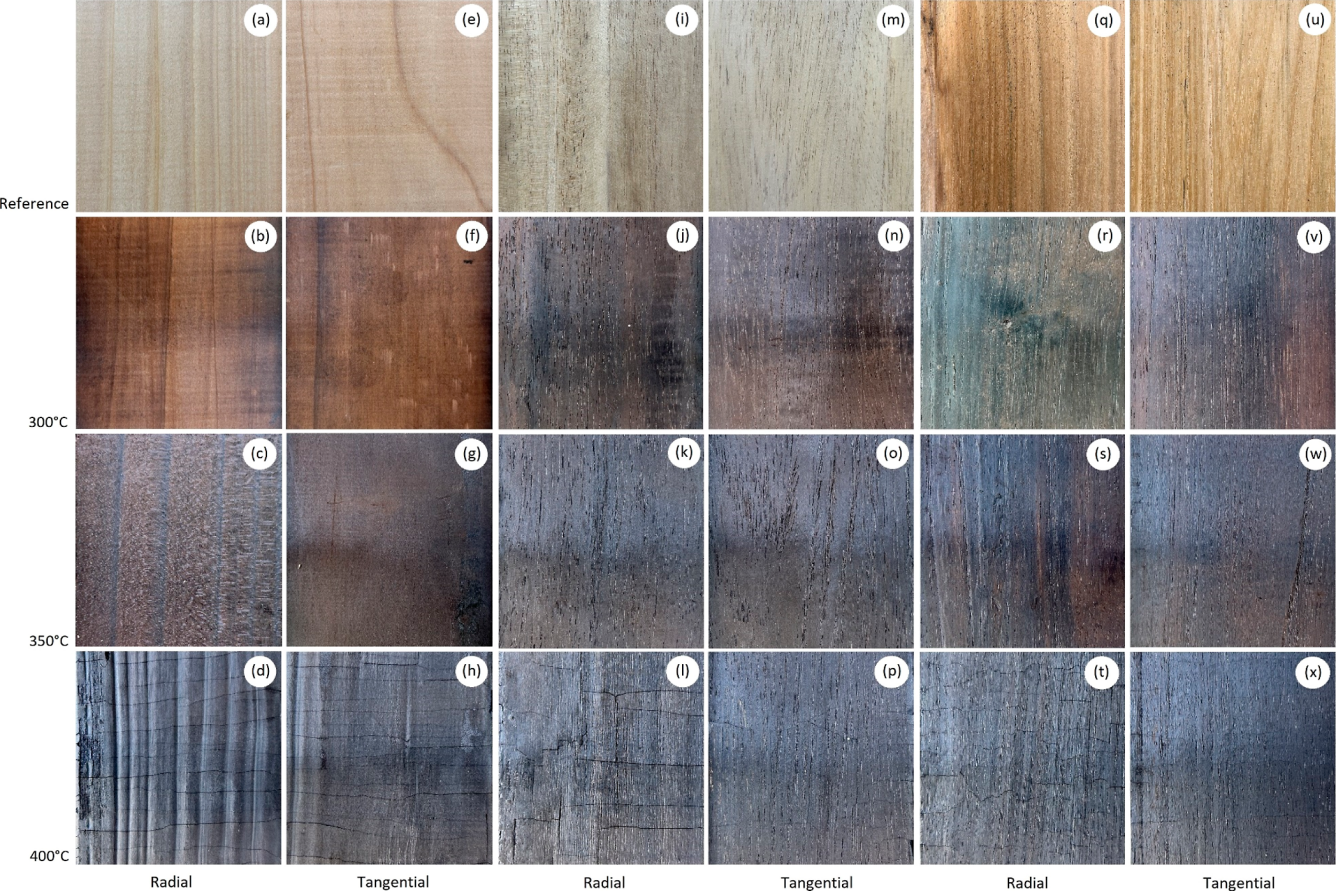
Charring surfaces of solid wood of *C. lusitanica* (a–h), *G. arborea* (i–p), and *T. grandis* (q–x) by temperature and grain orientation. Photograph of Carolina Tenorio Monge.

Figure 3 shows a sample of the reference and charring surfaces for the three species and the treatments evaluated. In general, the effect of the charring temperature was observed not only on the color of the wood but also on the quality of the surface. The samples charred at 300 and 350 °C did not present cracks on the surface, while in the samples at 400 °C, cracks and splits were observed on the surface, where in some cases they also presented detachments from the charring surface, mainly in the wood of *C. lusitanica* and *G. arborea* (Figure 3).

Because of the one-sided charred surface process, a change was observed in the color parameters of the woods of the three species. The three parameters L*, a*, and b* showed a decrease after the process, and the effect was greater as the charring temperature increased (Table 3). Some statistical differences were observed at the grain orientation level. For *C. lusitanica,* differences were observed at the temperature of 300 °C for the parameters a* after and b* before charring and at the temperature of 400 °C for the parameters L* and a* before charring (Table 3). For *G. arborea,*differences were only observed at the temperature at 400 °C for the L* parameter after charring, and in the case of *T. grandis, differences were observed* in the L* and a* parameters after charring at 350 °C treatments (Table 3).

**Table 3:** Color Parameters in the Surface of Radial and Tangential Wood of *C. lusitanica*, *G. arborea,* and *T. grandis* before and after Charring^a^

specie	temperature	grain orientation	L* parameter		a* parameter		b* parameter	
			before	after	before	after	before	after
*C. lusitanica*	300 °C	radial	71.7^A^	33.5^A^	11.5^A^	7.8^A^	27.4^A^	12.7^A^
		tangential	70.8^A^	31.2^A^	11.7^A^	6.5^B^	25.8^B^	10.8^A^
	350 °C	radial	71.7^A^	24.8^A^	10.1^A^	2.3^A^	25.5^A^	2.2^A^
		tangential	70.9^A^	24.7^A^	11.3^A^	2.9^A^	27.2^A^	3.1^A^
	400 °C	radial	72.1^A^	24.6^A^	10.1^B^	1.1^A^	25.6^A^	0.6^A^
		tangential	69.0^B^	24.7^A^	12.1^A^	1.0^A^	26.4^A^	0.3^A^
*G. arborea*	300 °C	radial	70.5^A^	25.9^A^	3.7^A^	3.7^A^	21.0^A^	5.3^A^
		tangential	67.4^A^	24.3^A^	4.7^A^	3.7^A^	22.4^A^	5.2^A^
	350 °C	radial	64.3^A^	23.1^A^	4.5^A^	1.3^A^	20.6^A^	1.5^A^
		tangential	65.0^A^	22.7^A^	5.1^A^	1.8^A^	21.5^A^	2.6^A^
	400 °C	radial	71.6^A^	24.9^A^	4.0^A^	0.8^A^	22.3^A^	1.3^A^
		tangential	69.0^A^	23.3^B^	4.7^A^	0.8^A^	22.7^A^	1.5^A^
*T. grandis*	300 °C	radial	59.5^A^	26.1^A^	10.5^A^	4.7^A^	27.7^A^	6.0^A^
		tangential	57.1^A^	24.5^A^	10.4^A^	4.2^A^	28.1^A^	4.9^A^
	350 °C	radial	59.6^A^	24.2^A^	10.7^A^	2.0^A^	27.6^A^	2.0^A^
		tangential	58.6^A^	22.3^B^	10.8^A^	1.3^B^	27.3^A^	1.1^A^
	400 °C	radial	60.3^A^	24.8^A^	9.9^A^	0.4^A^	28.6^A^	0.2^A^
		tangential	58.4^A^	24.9^A^	11.0^A^	0.4^A^	28.2^A^	0.3^A^

a Note: different letters for each parameter represent statistical differences between the grain orientation (radial and tangential) for each temperature (significance of 0.05).

**Figure 4:**
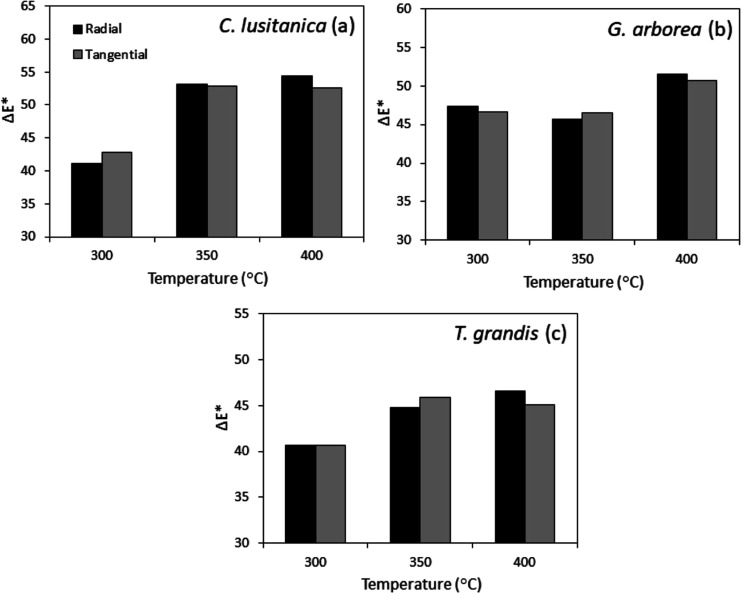
Color change in the surface charring wood of *C. lusitanica* (a), *G. arborea* (b), and *T. grandis* (c).

Regarding the color change (ΔE*) of the wood after the process, no statistical differences were observed in the temperatures evaluated at the grain orientation level for any of the three species; however, it was observed that *C. lusitanica* wood had the highest ΔE*, followed by *G. arborea* and *T. grandis* (Figure 4). For *C. lusitanica* and *T. grandis,* the greatest increase in the value of ΔE* was from 300 to 350 °C, while from 350 to 400 °C, the change in the value of ΔE* was minimal (Figure 4a,c). In *G. arborea*, the greatest change in the ΔE* value was from 350 to 400 °C, and from 300 to 350 °C, the ΔE* value was similar (Figure 4b).

### Sorption Characteristics

**Figure 5:**
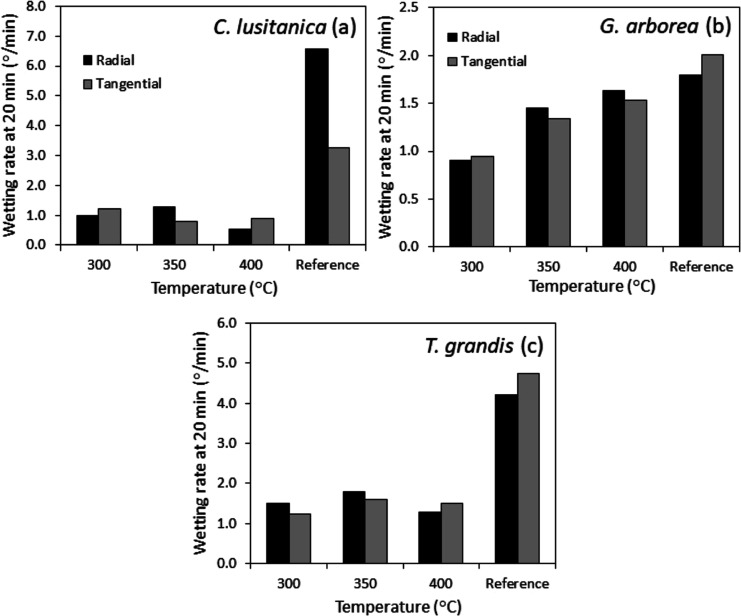
Wetting rate in the reference and charring surfaces of radial and tangential wood of *C. lusitanica* (a), *G. arborea* (b), and *T. grandis* (c).

**Figure 6:**
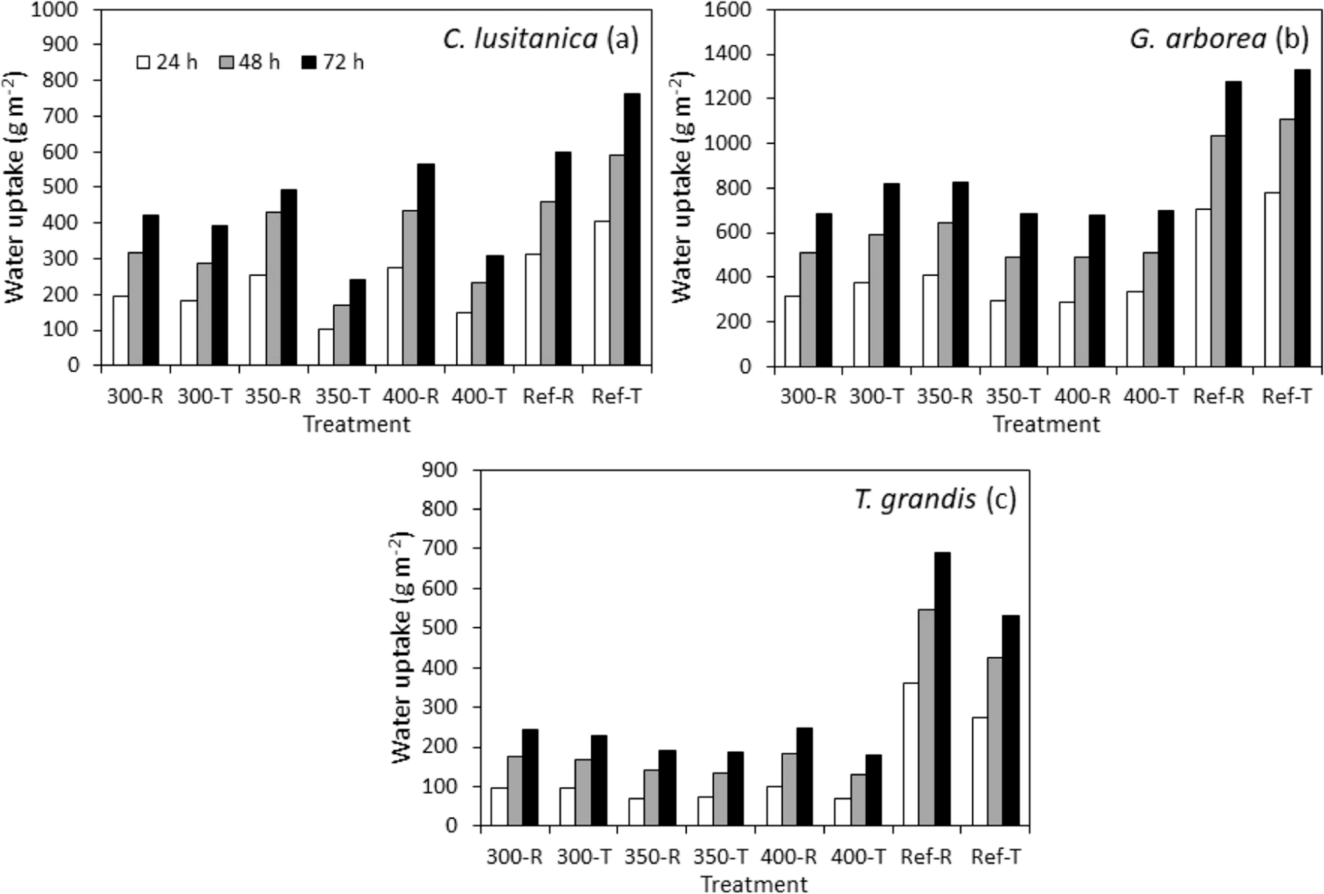
Water uptake for the reference and charring surface of radial and tangential wood of *C. lusitanica* (a), *G. arborea* (b), and *T. grandis* (c).

An effect was observed in the samples with the one-sided charred surface related to the reference in terms of the wetting rate values obtained for *C. lusitanica* and *T. grandis*. For both species and in all the treatments evaluated, the values of the wetting rate were significantly lower than those obtained in reference samples (Figure 6a,c). In *G. arborea,* the difference between the reference samples and the one-sided charred surface samples is low (Figure 6b). *C. lusitanica* was the species that presented the lowest values of the wetting rate for the charred specimens, followed by *G. arborea* and *T. grandis* (Figure 6). Some differences at the level of grain orientation can be observed in the temperatures evaluated for the three species; however, it is not possible to establish any pattern (Figure 6). For *C. lusitanica,* the treatment of charring at 400 °C presented the lowest values, for *G. arborea,* this was for the samples charred at 300 °C, while for *T. grandis, it was for* the samples charred at 300 and 400 °C (Figure 5).

Regarding water uptake, the effect of the one-sided charred surface related to the reference was observed for the three species where no treatment presented higher values than the reference at 24, 48, and 72 h (Figure 6). However, this effect was more marked for *G. arborea* and *T. grandis*, and for *C. lusitanica,* the difference between the reference and the one-sided charred surface samples is low (Figure 6a). Also, it was observed that *G. arborea* was the species that presented the highest values of water uptake at 72 h in all of the treatments evaluated, followed by *C. lusitanica* and *T. grandis* (Figure 6). For *C. lusitanica* and *T. grandis,* it was observed that the radial samples presented relatively higher averages in relation to the tangential samples; for *G. arborea,* this was only true at the temperature of 350 °C (Figure 6). Furthermore, for *C. lusitanica* and *T. grandis,* the treatment that presented the lowest average water uptake was 400-T, while for *G. arborea,* it was 400-R (Figure 6).

## Discussion

### Physical Characteristics

The loss of density that the wood samples of the three species had is due to the one-sided charred surface process. Although this loss of density was observed in all the treatments evaluated, there were no differences at the level of grain orientation, but the effect of the temperatures applied in the process is evident; specifically, the 400 °C samples presented the greatest decrease in density for the three species (Table 2 and Figure 4). This same behavior was observed at the Δ*t*hickness level (Table 2). This decrease in the density and thickness of the samples during the one-sided surface charring process is associated with a series of intercalated thermal degradation reactions of the polymers that form the wood.^27^ Hemicelluloses exhibit low thermal stability compared to cellulose, whereas lignin possesses high structural diversity and degrades gradually over a wide temperature range compared to carbohydrates.^28−30^ However, it must be understood that these polymers have different decomposition temperatures, where degradation of hemicelluloses starts at 180 °C or less,^31^ lignin degrades from 250 to 450 °C,^32,33^ and the minimum temperature for decomposition of cellulose crystals varies between 300 and 360 °C.^34^ The depolymerization of cellulose, production of volatile compounds, formation of oxidation products, and charring result at a temperature of approximately 300 °C. As the temperature increases, the degree of polymerization of cellulose decreases and the crystallinity increases.^35^

This might be due to the preferred degradation of the less ordered molecules during the thermal treatment.^36^ So, samples charred with the lowest temperature (300 °C) will present a lower loss of density and thickness in relation to samples charred at 400 °C because when the wood is subjected to high temperatures, there is a greater degradation of its three polymers (hemicellulose, cellulose, and lignin), while at low temperatures, the degradation is less. Another effect of the degradation of wood polymers at high temperatures is the presence of cracks and splits on the surface or, in some cases, the presence of detachments from the charring surface (Figure 3), a product precisely of depolymerization of cellulose and lignin.

In this study, *G. arborea* is the species that presented the greatest loss of density (approximately 10.4%) and the highest average charring thickness after the one-sided charred surface process (Table 2). This difference in density losses between the different species can be explained by considering two parameters of wood: its density and thermal conductivity. According to Friquin,^37^ the temperature range in which wood begins to decompose, starting with hemicelluloses, depends on the heating rate, species, density, or moisture content. Bartlett et al.^38^ point out that higher density species will generally char more slowly due to the greater mass of material to pyrolyze; thus, more energy is required to fuel this endothermic process. In this study, *G. arborea* has a density of 0.45 g/cm^3^, lower than that of *C. lusitanica* with 0.51 g/cm^3^ and *T. grandis* with 0.62 g/cm^3^ (Table 2). So, it was expected that *G. arborea*, which presented the lowest density, would also present a greater decrease in density because the heat supplied will heat less mass in relation to *C. lusitanica* and *T. grandis* species with higher density that require greater energy to achieve carbonization. Also, species with lower density typically had lower thermal conductivity, thus resulting in a faster temperature increase at the surface, thus pyrolyzing and charring earlier,^38^ which means that *G. arborea* chars more quickly than the other two species.

In the curves of the density profiles of the one-sided charred surface, it is possible to observe the effect of the temperature applied in the process and the grain orientation of the samples (Figure 2). Regarding temperature, as mentioned previously, the higher the process temperature, the greater the degree of degradation of the wood components; hence, in the 400 °C curves, a greater loss of density is observed on the charred side (Figure 2). Regarding the grain orientation, the radial samples present more uniform curves (Figure 2a,c,e), while the tangential samples present a greater variation in the density curves for the three species (Figure 2b,d,f). This variation between the radial and tangential samples is due to the different anatomical characteristics in the cross-sections of the wood of the three species. For *C. lusitanica*, being a conifer, the differences between orientations are due to the marking of the growth ring in the cross sections. In the samples with radial orientation, the effect of the growth rings is not observed, so the density profiles are more homogeneous (Figure 2a). Contrary to samples with tangential orientation, where the effect of growth rings is observed, more heterogeneous profiles are produced due to density changes between early wood and late-wood wood (Figure 2b). According to Moya et al.^22^*G. arborea* presents diffuse, or semiannular, porosity, in addition to presenting vessels with average diameters of 189 μm, which produces a marked difference between the radial and tangential orientations, with the tangential orientation being less uniform because of the difference of vessel size due to this type of porosity (Figure 2c,d). *T. grandis* only presents semiannular porosity,^22^ which causes samples with tangential orientation to present less uniform density profiles, as mentioned above due to the difference in vessel size typical of semiannular porosity (Figure 2e,f).

### Quality of One-Sided Surface Charred Wood

Color in the reference wood is related with extractives and polymers presence in the cell wall.^39,40^ It was found that the redness (a*) and luminosity (L*) parameters correlate highly with the extractive content of wood, while the yellow color parameter is correlated with the photochemistry of cell wall chemical components (cellulose, hemicellulose, and lignin). So, reference wood is common that presented high values of L* parameters (values from 35 to 80), with tonalities of readiness (a* values from 5 to 20) and yellowness (b* values from 10 to 35).^41^ In this study, the values found in the reference wood for the three species agreed with this range in all parameters. However, during the surface charring process, there are many chemical changes in wood, which affect color parameters. In general, there is a decrease of all color parameters (Table 3), the greatest decrease being for L* parameters, followed by b* parameters and a* parameters having the lowest decrease (Table 3).

In the process of surface charring, temperature affects the way polymers decompose in wood and therefore affects charred surface color, as presented in Figure 3. Depending on the temperature used in the charring surface process (300, 350, and 400 °C), hemicelluloses degrade through different reactions such as oxidation, dehydration, decarboxylation, and hydrolysis,^42^ which contributes to the decrease of color parameters (Table 3), changing the color of the reference to a dark color on the charred surface. Cellulose and lignin decomposition contributed probably in greater proportion to the color change, where the decomposition of both polymers produces vapors and gases or volatile contents such as CO_2_, CO, H_2_, and water, a product of the elimination by processes of oxidation, dehydration, decarboxylation, and hydrolysis of hydrogen and oxygen. In addition, the fixed carbon content increases and the color of the surface acquires dark tones.^43^ However, the decomposition of cellulose and lignin occurs in a wide range of temperatures (375–500 °C), and in this study, with the lowest temperature (300 °C), cellulose and lignin may show less decomposition in relation to that at the temperatures of 350 and 400 °C, causing the color change (ΔE*) to present the lowest values in the three species (Figure 4). On the contrary, with the highest temperature (400 °C), cellulose and lignin decomposed, causing the surface of the wood to have a dark color, and the ΔE* values were the highest (Figure 4b).

Another important aspect to mention is that the color change values (ΔE*) cannot be used to determine the degree of charred surface because this parameter is related to the initial color of the wood (reference). One objective of the carbonization process is to darken the surface of the wood, so all species will tend to reach a dark color, but some species will present reddish or brown tones since they have lower L* values and higher a* values than the species with light tones. And when the ΔE* is calculated, the values become lower because they presented the lowest values of the L* parameter, as is the case of *T. grandis* in this study, which presented the lowest values of ΔE* as a product because the parameter L* was lower in relation to the value of this parameter in *C. lusitanica* and *G. arborea* (Table 3).

### Sorption Characteristics

It is possible to affirm that the one-sided charred surface process increased the hydrophobicity of wood of the three species in relation to the reference samples, as could be observed in the results obtained from the wetting rate and water uptake (Figures 5 and 6). Many studies point out the effect that temperature has on the hydrophobicity of wood: Šeda et al.^44^ point out that the hydrophobicity of heat-treated wood can be explained by the degradation of the amorphous zones of the chemical components of the wood, which leads to an increase in the crystallinity of cellulose because of temperature since sorption of water by the amorphous region is higher than for crystalline cellulose. Furthermore, Šernek et al.^45^ indicate that higher amounts of nonpolar components on the surface of samples exposed to higher temperatures cause an increase in hydrophobicity, which is attributed to extractive migrations and deposition of volatile organic compounds. And according to Lopes et al.,^46^ the dehydration reactions of hemicelluloses that are ongoing during heat treatment can degrade OH groups, which causes a decrease in water penetration into the wood surface.

In the case of *C. lusitanica* and *T. grandis,* the samples charred at any temperature and grain orientation presented a successful chemical modification in terms of the wetting rate of wood (Figure 5a,c). Čermák et al.^20^ point out that there are differences at the level of grain orientation in terms of sorption characteristics, that the charring increased the hydrophobic behavior more in the radial specimens of beech wood. But for Kocaefe et al.,^47^ the differences in radial and tangential directions are not significant for heat-treated white ash and soft maple. This latest research is in line with our results, in which we could not establish a pattern in terms of grain orientation for the three species evaluated (Figure 5). In the case of *G. arborea*, although the wetting rate values of the one-sided charred surface samples are low (<1.7°/min), for the charred treatments at 350 and 400 °C, the values are closer to the reference (Figure 5b), which could be related to the fact that *G. arborea* chars faster than the other two species, which was verified with the loss of density (Table 2) that the samples of this species presented after the process and the formation of cracks on the charred surface (Figure 2). According to Blankenhorn,^48^ the charred surface layer is hydrophobic, cross-linked, and aromatic but also porous and brittle, especially at temperatures above 300 °C, so when the capillary absorption takes place in a cracked surface, this may lead to water penetrating the unmodified inner wood,^7^ causing the wetting rate values of these treatments to be like those obtained in the reference. In addition, the reference samples of *G. arborea* presented the lower wetting rate values, which could be a consequence of the fact that this species is characterized by presenting tyloses inside the vessels,^22^ which makes the capillary absorption low.

Regarding water uptake, the effect of the one-sided charred surface was observed for the three species where no treatment presented higher values than the reference (Figure 6). These results seem to indicate that chemical modification took place at the level of hemicelluloses and amorphous areas of cellulose. According to Šeda et al.,^44^ the reduced water absorption is likely to be due to a reduction in the number of hydroxyl groups (−OH) in the celluloses and hemicelluloses because of high temperature treatment. Furthermore, the decreased accessibility of water molecules to cellulose hydroxyl groups due to the increase in cellulose crystallinity and cross-linking in lignin can also play an important role.^49,50^ However, the results indicate that the modification was greater in the case of *G. arborea* and *T. grandis*, where the differences of the one-sided charred surface with the reference samples was greater (Figure 6b,c). Softwood species have a higher proportion of lignin than hardwood species, and during carbonization, the aromatic carbon present in lignin is lost at temperatures above 350 °C,^3^ which makes the charred surface brittle, increasing water absorption and porosity.^51^ Therefore, this increase in porosity provides a better path for water to enter the wood and could be the reason why *C. lusitanica* presents fewer differences with the reference wood in relation to *T. grandis* and *G. arborea*.

*G. arborea* is the species that presented the highest water uptake values for both the reference and one-sided charred surface samples (Figure 6b). In the case of water uptake, the movement of water within the wood occurred not by capillary forces but by diffusion, which means that the permeability and porosity of each species influences the results obtained.^3^ It is possible to indicate that the intrinsic characteristics of each species, especially related to the size of their anatomical structures, influence their permeability. *G. arborea* has vessels with a diameter of 189 μm, larger than those of *T. grandis,* which have a diameter of 150 μm,^22^ and this makes the permeability of *G. arborea* greater since it is expected for the movement and speed of water to be faster in species with larger diameter pores. In the case of soft woods, such as *C. lusitanica*, their anatomical structure is mainly composed of tracheids, and they are also characterized by having a higher proportion of lignin in relation to hardwood species. This characteristic causes the charred surface of *C. lusitanica* to present greater porosity^51^ and therefore greater water uptake in relation to *T. grandis*, but not greater than *G. arborea* (Figure 6).

Furthermore, it was observed for *C. lusitanica* that the radial samples tended to present slightly higher values than the tangential samples, especially at temperatures of 350 and 400 °C (Figure 6a), a situation that did not occur in *T. grandis* and *G. arborea* (Figure 6b,c). Again, these differences are attributed to the anatomical structure of each species. Softwoods, such as *C. lusitanica*, have tracheid pits, which are mostly associated with the radial side of the fibers.^52^ This means that the samples with radial orientations will present a greater number of micropores (greater porosity) than the samples with tangential orientations; this will allow for greater permeability or diffusivity of water on the surface charring, increasing the values of water uptake in the radial orientations (Figure 6a).

## Conclusions

In conclusion, we successfully developed the one-sided surface charring process for wood samples of *C. lusitanica*, *G. arborea,* and *T. grandis*. The effect of the applied temperature was greater than the effect of the grain orientation of the samples after the process. The only effect of the grain orientation was in the density profiles, where the radial density profiles were more homogeneous in relation to the tangential ones because of the anatomical structure of each species. Temperature had a greater effect on the changes in density and color observed; samples charred at 300 °C presented a lower loss of density and thickness in relation to samples charred at 400 °C because of the greater degradation of its three polymers (hemicellulose, cellulose, and lignin). These changes in the chemical structure of the wood also caused a decrease of all color parameters (L*, a*, and b*), the greatest decrease being in the samples charred at 400 °C for the three species. Another effect of the applied temperature is observed in the presence of cracks and splits on the surface, or in some cases the presence of detachments from the charring surface, especially in the samples charred at 350 and 400 °C, a product of depolymerization of cellulose and lignin.

One-sided surface charring reduced the water sorption of the wood samples in comparison to reference samples, especially for *C. lusitanica* and *T. grandis*. The chemical changes produced in the charred surface wood (thermal degradation of the polymers that form the wood, the movement of extractives, and the reduction of OH groups and polar components, among others) caused along with the changes to the anatomical structure to each species changes in the sorption properties of the charred wood due to the application of high temperatures. *G. arborea* was the species that had the greatest influence of its initial density and anatomical structure on its behavior during the charring process and in the changes observed in the charred surfaces properties. It was observed that *G. arborea* chars faster than *C. lusitanica* and *T. grandis*, which caused a greater loss of density and thickness; in addition, it presented wetting rate values like those of the reference and the highest water uptake values.

In general, for *C. lusitanica* and *T. grandis*, the samples carbonized at 350 °C presented the best results, in terms of the quality of the carbonized surface and increasing hydrophobicity. The wood of *G. arborea* tends to char more quickly than the other two species due to the intrinsic characteristics of the species; this affects its performance, especially in terms of the quality of the charred surface (cracks and splits), affecting the results of the water uptake and wetting rate, so for this species, the ideal is to work with slightly lower temperatures, between 300 and 350 °C.

## Data Availability

The data sets used during the current work are available from the corresponding author upon request and in the Knowledge Network for Biocomplexity (KNB) at 10.5063/F13N21WR.

## References

[ref1] Hill C.; Kymäläinen M.; Rautkari L. Review of the use of solid wood as an external cladding material in the built environment. J. Mater. Sci. 2022, 57, 9031–9076. 10.1007/s10853-022-07211-x.

[ref2] Khademibami L.; Bobadilha G. S. Recent Developments Studies on Wood Protection Research in Academia: A Review. Front. For. Glob. Change 2022, 5, 793177 10.3389/ffgc.2022.793177.

[ref3] Kymäläinen M.; Turunen H.; Čermák P.; Hautamäki S.; Rautkari L. Sorption-Related Characteristics of Surface Charred Spruce Wood. Materials 2018, 11, 2083 10.3390/ma11112083. 30355998 PMC6266808

[ref4] Kropat M.; Hubbe M. A.; Laleicke F. Natural, accelerated, and simulated weathering of wood: A review. BioResources 2020, 15 (4), 9998–10062. 10.15376/biores.15.4.Kropat.

[ref5] Morozovs A.; Laiveniece L.; Lubinskis V. Wood one-side surface charring of timber for claddings or recycled wood. International Scientific Conference Engineering for Rural Development. Jelgava,May 26–28; Latvia Univ. of Life Sciences and Technologies, 2021.

[ref6] Čermák P.; Vahtikari K.; Rautkari L.; Laine K.; Horáček P.; Baar J. The effect of wetting cycles on moisture behaviour of thermally modified Scots pine (Pinus sylvestris L.) wood. J. Mater. Sci. 2016, 51, 1504–1511. 10.1007/s10853-015-9471-5.

[ref7] Kymäläinen M.; Hautamäki S.; Lillqvist K.; Segerholm K.; Rautkari L. Surface modification of solid wood by charring. J. Mater. Sci. 2017, 52, 6111–6119. 10.1007/s10853-017-0850-y.

[ref8] Wentzel M.; Altgen M.; Militz H. Analyzing reversible changes in hygroscopicity of thermally modified eucalypt wood from open and closed reactor systems. Wood Sci. Technol. 2018, 52, 889–907. 10.1007/s00226-018-1012-3.

[ref9] Sandberg D.; Kutnar A.; Karlsson O.; Jones D. Wood Modification Technologies. Principies, Sustainability, and the Need for Innovation; CRC Press, 2021; .

[ref10] Esteves B. M.; Pereira H. M. Wood modification by heat treatment: A review. Bioresources 2008, 4, 370–404. 10.15376/biores.4.1.esteves.

[ref11] Zelinka S. L.; Altgen M.; Emmerich L.; Guigo N.; Keplinger T.; Kymäläinen M.; Thybring E. E.; Thygesen L. G. Review of wood modification and wood functionalization technologies. Forests 2022, 13 (7), 1004 10.3390/f13071004.

[ref12] Rowell R. M. Understanding wood surface chemistry and approaches to modification: A review. Polymers 2021, 13 (15), 2558 10.3390/polym13152558. 34372161 PMC8348385

[ref13] Lin S.; Qin Y.; Huang X.; Gollner M. Use of pre-charred surfaces to improve fire performance of wood. Fire Saf. J. 2023, 136, 103745 10.1016/j.firesaf.2023.103745.

[ref14] Bekhta P.; Niemz P. Effect of high temperature on the change in color, dimensional stability and mechanical properties of spruce wood. Holzforschung 2003, 57 (5), 539–546. 10.1515/HF.2003.080.

[ref15] Li G.; Gao L.; Liu F.; Qiu M.; Dong G. Quantitative studies on charcoalification: Physical and chemical changes of charring wood. Fundam. Res. 2024, 4, 113–122. 10.1016/j.fmre.2022.05.014. 38933840 PMC11197656

[ref16] Soytürk E. E.; Kartal S. N.; Arango R. A.; Ohno K. M.; Solhan E.; Çağlayan İ.; Ibanez C. M. Surface carbonization of wood: comparison of the biological performance of Pinus taeda and Eucalyptus bosistoana woods modified by contact charring method. Wood Mater. Sci. Eng. 2023, 18 (6), 1888–1899. 10.1080/17480272.2023.2198993.

[ref17] Xian-jun L. Effect of high temperature heat treatment on XRD properties of Chinese fir wood. For. Mach. Woodwork. Equip. 2009, 37 (2), 25–28.

[ref18] Ebner D.; Stelzer R.; Barbu M. C. Study of wooden surface carbonization using the traditional Japanese Yakisugi technique. Pro Ligno 2019, 15 (4), 278–283.

[ref19] Kymäläinen M.; Dömény J.; Rautkari L. Moisture Sorption of Wood Surfaces Modified by One-Sided Carbonization as an Alternative to Traditional Façade Coatings. Coatings 2022, 12, 1273 10.3390/coatings12091273.

[ref20] Čermák P.; Dejmal A.; Paschová Z.; Kymäläinen M.; Dömény J.; Brabec M.; Hess D.; Rautkari L. One-sided surface charring of beech wood. J. Mater. Sci. 2019, 54, 9497–9506. 10.1007/s10853-019-03589-3.

[ref21] Méndez-Mejías L. D.; Moya R. Effect of thermo-treatment on the physical and mechanical, color, fungal durability of wood of Tectona grandis and Gmelina arborea from forest plantations. Mater. Sci. 2018, 24 (1), 59–68. 10.5755/j01.ms.24.1.17545.

[ref22] Moya R.; Tenorio C.; Salas C.; Berrocal A.; Muñoz F. Tecnología de la madera de Plantaciones forestales de Costa Rica; Editorial Tecnológica de Costa Rica. Editorial de la Universidad de Costa, 2019.

[ref23] Tenorio C.; Moya R. Development of a Thermo-Hydro-Mechanical Device for Wood Densification Adaptable to Universal Testing Machines and Its Evaluation in a Tropical Species. J. Test. Eval. 2021, 49, 2597–2608. 10.1520/JTE20180760.

[ref24] Gaitan-Alvarez J.; Berrocal A.; Lykidis C.; Moya R.; Mantanis G. Furfurylation of tropical wood species with and without silver nanoparticles. Part II. Evaluation of wood properties. Wood Mater. Sci. Eng. 2023, 18 (1), 112–119. 10.1080/17480272.2021.1992795.

[ref25] ASTM International. Standard Test Methods for Direct Moisture Content Measurement of Wood and Wood-Based Materials; ASTM D4442–20: West Conshohocken, PA, 2020.

[ref26] Bachmann J.; Horton R.; Van Der Ploeg R. R.; Woche S. Modified sessile drop method for assessing initial soil-water contact angle of sandy soil. Soil Sci. Soc. Am. J. 2000, 64 (2), 564–567. 10.2136/sssaj2000.642564x.

[ref27] Hakkou M.; Pétrissans M.; Zoulalian A.; Gérardin P. Investigation of wood wettability changes during heat treatment on the basis of chemical analysis. Polym. Degrad. Stab. 2005, 89, 1–5. 10.1016/j.polymdegradstab.2004.10.017.

[ref28] Beall F. C. Thermal degradation of wood. In Encyclopedia of Materials Science and Engineering; Bever M. B., Ed.; Pergamon Press, 1986.

[ref29] Cerc Korošec R.; Lavrič B.; Rep G.; Pohleven F.; Bukovec P. Thermogravimetry as a possible tool for determining modification degree of thermally treated Norway spruce wood. J. Therm. Anal. Calorim. 2009, 98, 189–195. 10.1007/s10973-009-0374-z.

[ref30] Helsen L.; Van den Bulck E. J. Kinetics of the low-temperature pyrolysis of chromated copper arsenate-treated wood. J. Anal. Appl. Pyrol. 2000, 53, 51–79. 10.1016/S0165-2370(99)00050-9.

[ref31] Sivonen H.; Maunu S. L.; Sundholm F.; Jamsa S.; Viitaniemi P. Magnetic resonance studies of thermally modified wood. Holzforschung 2002, 56, 648–654. 10.1515/HF.2002.098.

[ref32] Williams P. T.; Besler S. Thermogravimetric analyses of the components of biomass. In Advances in Thermochemical Biomass Conversion; Bridgwater A. V., Ed.; Blackie Academic & Professional, 1994; Vol. 2.

[ref33] Alén R.; Rytkönen S.; McKeough P. Thermogravimetric behavior of black liquors and their organic-constituents. J. Anal. Appl. Pyrolysis 1995, 31, 1–13. 10.1016/0165-2370(94)00811-E.

[ref34] Kim D. Y.; Nishiyama Y.; Wada M.; Kuga S.; Okano T. Thermal decomposition of cellulose crystallites in wood. Holzforschung 2001, 55, 521–524. 10.1515/HF.2001.084.

[ref35] Romagnoli M.; Vinciguerra V.; Silvestri A. Heat treatment effect on lignin and carbohydrates in Corsican pine earlywood and latewood studied by PY-GC-MS technique. J. Wood Chem. Technol. 2018, 38, 57–70. 10.1080/02773813.2017.1372479.

[ref36] Bhuiyan M. T. R.; Hirai N.; Sobue N. Changes of crystallinity in wood cellulose by heat treatment under dried and moist conditions. J. Wood Sci. 2000, 46, 431–436. 10.1007/BF00765800.

[ref37] Friquin K. L. Material properties and external factors influencing the charring rate of solid wood and glue-laminated timber. Fire Mater. 2011, 35 (5), 303–327. 10.1002/fam.1055.

[ref38] Bartlett A. I.; Hadden R. M.; Bisby L. A. A Review of Factors Affecting the Burning Behaviour of Wood for Application to Tall Timber Construction. Fire Technol. 2019, 55, 1–49. 10.1007/s10694-018-0787-y.

[ref39] Gierlinger N.; Jacques D.; Grabner M.; Wimmer R.; Schwanninger M.; Rozenberg P.; Pâques L. E. Colour of larch heartwood and relationships to extractives and brown-rot decay resistance. Trees 2004, 18, 102–108. 10.1007/s00468-003-0290-y.

[ref40] Moya R.; Fallas R. S.; Bonilla P. J.; Tenorio C. Relationship between wood color parameters measured by the CIELab system and extractive and phenol content in Acacia mangium and Vochysia guatemalensis from fast-growth plantations. Molecules 2012, 17 (4), 3639–3652. 10.3390/molecules17043639. 22450677 PMC6268558

[ref41] Nishino Y.; Janin G.; Chanson B.; Détienne P.; Gril J.; Thibaut B. Colorimetry of wood specimens from French Guiana. J. Wood Sci. 1998, 44, 3–8. 10.1007/BF00521867.

[ref42] Kocaefe D.; Poncsak S.; Boluk Y. Effect of Thermal Treatment on The Chemical Composition and Mechanical Properties of Birch and Aspen. BioResources 2008, 3, 517–537. 10.15376/biores.3.2.517-537.

[ref43] Gospodinova D.; Dineff P. New Surface Charred Wood Effects for Charcoal Coating, Graphic Image and Drawing. 11th Electrical Engineering Faculty Conference (BulEF); IEEE: Varna, Bulgaria, 2019; pp 1–9.

[ref44] Šeda V.; Machová D.; Dohnal J.; Dömény J.; Zárybnická L.; Oberle A.; Vacenovská V.; Čermák P. Effect of One-Sided Surface Charring of Beech Wood on Density Profile and Surface Wettability. Appl. Sci. 2021, 11, 4086 10.3390/app11094086.

[ref45] Šernek M.; Kamke F. A.; Glasser W. G. Comparative analysis of inactivated wood surfaces. Holzforschung 2004, 58, 22–31. 10.1515/HF.2004.004.

[ref46] Lopes J. D. O.; Garcia R. A.; Nascimento A. M. d. Wettability of the Surface of Heat-Treated Juvenile TeakWood Assessed by Drop Shape Analyzer. Maderas Cienc. tecnol. 2018, 20, 249–256. 10.4067/S0718-221X2018005002801.

[ref47] Kocaefe D.; Poncsak S.; Doré G.; Younsi R. Effect of Heat Treatment on the Wettability of White Ash and Soft Maple by Water. Holz als Roh- Werkst. 2008, 66, 355–361. 10.1007/s00107-008-0233-9.

[ref48] Blankenhorn P. R.; Kline D. E.; Beall F. C. Dynamic mechanical behavior of carbonized black cherry wood (prunus serotina ehrh.). Carbon 1973, 11, 603–611. 10.1016/0008-6223(73)90326-6.

[ref49] Boonstra M.; Tjeerdsma B. Chemical analysis of heat treated softwoods. Holz als Roh- Werkst. 2006, 64, 204–211. 10.1007/s00107-005-0078-4.

[ref50] Hill C. Wood Modification Chemical, Thermal and other Processes; Wiley, 2006.

[ref51] Keech O.; Carcaillet C.; Nilsson M. C. Adsorption of allelopathic compounds by wood-derived charcoal: the role of wood porosity. Plant Soil 2005, 272, 291–300. 10.1007/s11104-004-5485-5.

[ref52] Esteban L. G.; de Palacios P.; Heinz I.; Gasson P.; García-Iruela A.; García-Fernández F. Softwood Anatomy: A Review. Forests 2023, 14 (2), 323 10.3390/f14020323.

